# AGING WITH MENTAL HEALTH CONDITONS AND MOTIVATION FOR SELF-CARE: THE HANDWASHING WORKSHOP

**DOI:** 10.1093/geroni/igad104.3031

**Published:** 2023-12-21

**Authors:** Aryane Cazumbá, Gabriela Bellizzi, Marcia Varricchio, Neide Verçosa, Felipe Paci Soares, Erica Paci Soares, Jaqueline Da Silva

**Affiliations:** UNIFASE, Petropolis, Rio de Janeiro, Brazil; UNIFASE / FMP, Petropolis, Rio de Janeiro, Brazil; Federal University of Rio de Janeiro (UFRJ), RIo de Janeiro, Rio de Janeiro, Brazil; Federal University of Rio de Janeiro (UFRJ), RIo de Janeiro, Rio de Janeiro, Brazil; Colégio Bom Jesus São José (CBJSJ), Petropolis, Rio de Janeiro, Brazil; Colégio Bom Jesus São José (CBJSJ), Petropolis, Rio de Janeiro, Brazil; Federal University of Rio de Janeiro (UFRJ), RIo de Janeiro, Rio de Janeiro, Brazil

## Abstract

In Brazil, data from the Influenza Epidemiological Surveillance Information System (SIVEP-Flu), indicate that up to June 2020, about 71% of deaths from Covid19 were of elderly, with and without comorbidities. Since the SARS-Cov2 pandemic, health of the elderly population with mental health problems has been hard hit. The study scenario was the 2023 Summer Edition of the Intergenerational and Inclusion Camp on Health, Science and Technology / Lab Project for the Valorization of Aging (PROVE). The implementation research design was a teaching-research-service action by graduate, undergraduate and two high school students. Supported by the lab faculty and librarian, students searched for evidence-based practices, and in the light of health equity, selected the hand washing demand of underserved older adults to develop a ludic-informative workshop, for motivating self-care practices. In the methodological trajectory, participants (i) were divided into two teams; (ii) had representatives put latex gloves and blindfolds on and washable colored paint poured on the gloves; (iii) received instructions to demonstrate how they washed their hands; (iv) blindfolds were removed and a debate took place on the procedure and its importance; (v) students demonstrated the correct technique using gloves and paint. Older adults reported having had fun and learning significantly more. The approach was evaluated as empowering, noting that the visualization of the inks on the gloves specifically allowed identifying points to be refined. Other topics and subsequent workshops were suggested. In conclusion, light and flexible fun, evidence-based and motivating strategies are protective and promote comprehensive health care.

